# Extracellular vesicles from T cells overexpress miR-146b-5p in HIV-1 infection and repress endothelial activation

**DOI:** 10.1038/s41598-019-44743-w

**Published:** 2019-07-16

**Authors:** Estelle Balducci, Aurélie S. Leroyer, Romaric Lacroix, Stéphane Robert, Dilyana Todorova, Stéphanie Simoncini, Luc Lyonnet, Corinne Chareyre, Olivia Zaegel-Faucher, Joëlle Micallef, Isabelle Poizot-Martin, Patrice Roll, Françoise Dignat-George

**Affiliations:** 1 Aix Marseille Univ, INSERM, C2VN, Marseille, France; 2 APHM, Hôpital La Conception, Laboratoire d’Hématologie et de biologie vasculaire, Marseille, France; 3 APHM, Hôpital Sainte-Marguerite, Service d’Immuno-hématologie clinique, Marseille, France; 4 APHM, Hôpital la Timone, Service de Pharmacologie, Marseille, France; 5 Aix Marseille Univ, CNRS, INT, Inst Neurosci Timone, Marseille, France; 6 Aix Marseille Univ, Inserm U912 (SESSTIM), Marseille, France; 7 Aix Marseille Univ, INSERM, MMG, Marseille, France; 8 APHM, Hôpital la Timone, Service de Biologie Cellulaire, Marseille, France

**Keywords:** Cell biology, Cell signalling

## Abstract

Human immunodeficiency virus type 1 (HIV-1) infection promotes a generalized activation of host responses that involves not only CD4 T cells, but also cells of the microenvironment, which are not directly infected, such as endothelial cells. The mechanisms triggering HIV-1-associated vascular alterations remain poorly understood. Extracellular vesicles (EVs), implicated in cell-to-cell communication, have been recently described as carriers of microRNAs (miRNAs). Here, we show that miR-146b-5p is upregulated in both CD4 T cells, CD4 T cell-derived EVs and circulating EVs obtained from antiretroviral therapy-naive HIV-1-infected patients. We further demonstrate that EVs from T cell line overexpressing miR-146b-5p mimics (miR-146b-EVs): 1) protect their miRNA cargo from RNase degradation, 2) transfer miR-146b-5p mimics into endothelial cells and 3) reduce endothelial inflammatory responses *in vitro* and *in vivo* in the lungs of mice through the downregulation of nuclear factor-κB-responsive molecules. These data advance our understanding on chronic inflammatory responses affecting endothelial homeostasis, in infectious and non-infectious diseases and pave the way for potential new anti-inflammatory strategies.

## Introduction

Extracellular vesicles (EVs) are a heterogeneous group of cell-derived membranous structures comprising exosomes, microvesicles (MVs) and apoptotic bodies, released by a large variety of cells^1^. Exosomes are vesicles characterized by a size ranging from 30 to 100 nm and the expression of tetraspanin proteins, ALIX, syntenin 1, and TSG101 but not phosphatidylserine (PS). They are formed through the fusion of multivesicular bodies with the plasma membrane and the subsequent release of intraluminal vesicles^2^. MVs are small elements ranging from 0.1 to 1 µm in diameter which result from the blebbing of cell membranes in response to cell activation or apoptosis. They generally express anionic phospholipid PS on the outer face of the membrane and membrane antigens representative of their parental cells^3^. Apoptotic bodies differ from other EVs by their larger size (>1 µm). They express PS and contain DNA and histones^4^. EVs are present in biological fluids and are involved in multiple physiological and pathological processes. EVs can be defined as circulating carriers of bioactive molecules displaying multiple functions in coagulation, fibrinolysis, inflammation, immune responses and angiogenesis^5–7^. During their biogenesis, EVs protectively package intracellular components such as proteins, lipids and genetic material and enable the transfer of biological information to target cells^2,8^. EVs have been described as carriers of microRNAs (miRNAs) implicated in cell-to-cell communication^9^. MiRNAs are small, non-coding RNAs containing 18–25 nucleotides, which are known to modulate gene expression by promoting the degradation of target mRNAs or by inhibiting their translation. Dysregulation of cellular miRNA expression has been associated with numerous pathologies, including human immunodeficiency virus type 1 (HIV-1) infection^10^. Interestingly, multiple cellular miRNAs have been shown to modulate both viral infectivity and replication by directly targeting the viral genome or by decreasing the expression of host proteins required for viral replication^11^.

There is now growing evidence that exosomes can mediate the transfer of miRNAs from virally-infected cells to uninfected bystander cells as demonstrated for Epstein-Barr virus infection^12^. This mechanism also potentially plays an important role in HIV-1 pathogenesis and control of host immune responses against infection. Furthermore, HIV-1 infection promotes a generalized activation of host responses that involve CD4 T cells and cells of the microenvironment, such as endothelial cells, which are not directly infected. However, the mechanisms triggering HIV-1-associated vascular alterations remain poorly understood.

In addition to the induction of chronic systemic inflammation and immune activation, HIV-1 infection has been associated with endothelial dysfunction resulting from the disruption of endothelial homeostatic responses^13^. Endothelial dysfunction has emerged as a pivotal mechanism involved in the increased risk of cardio-vascular diseases observed during HIV-1 infection in the absence or presence of suppressive antiretroviral therapy (ART)^14^. To our knowledge, little is known to date about the role played by EVs in endothelial dysfunction associated with HIV-1 infection.

In this study, we hypothesized that HIV-1 infection induces cellular miRNA expression in CD4 T cells, which may be carried by EVs and transferred in a paracrine manner to endothelial cells to regulate vascular homeostasis. Our findings demonstrate that miR-146b-5p is upregulated in both CD4 T cells and CD4 T cell-derived EVs in ART-naive HIV-1-infected patients. Using the CEM T cell line transfected with miR-146b-5p mimics, we demonstrated that EVs from CEM cells (CEM-EVs) overexpressing miR-146b-5p mimics (miR-146b-EVs) transfer this miRNA to endothelial cells *in vitro* and *in vivo*. This transfer leads to the inhibition of the nuclear factor-κB (NF-κB)-responsive genes, intercellular adhesion molecule 1 (ICAM-1) and vascular cell adhesion molecule 1 (VCAM-1), through the downregulation of interleukin-1 receptor-associated kinase 1 (IRAK1) and tumor necrosis factor-receptor-associated factor 6 (TRAF6).

## Results

### Biogenesis and characterization of CD4 T cell-derived EVs from ART-naive HIV-1-infected patients

**Figure 1:**
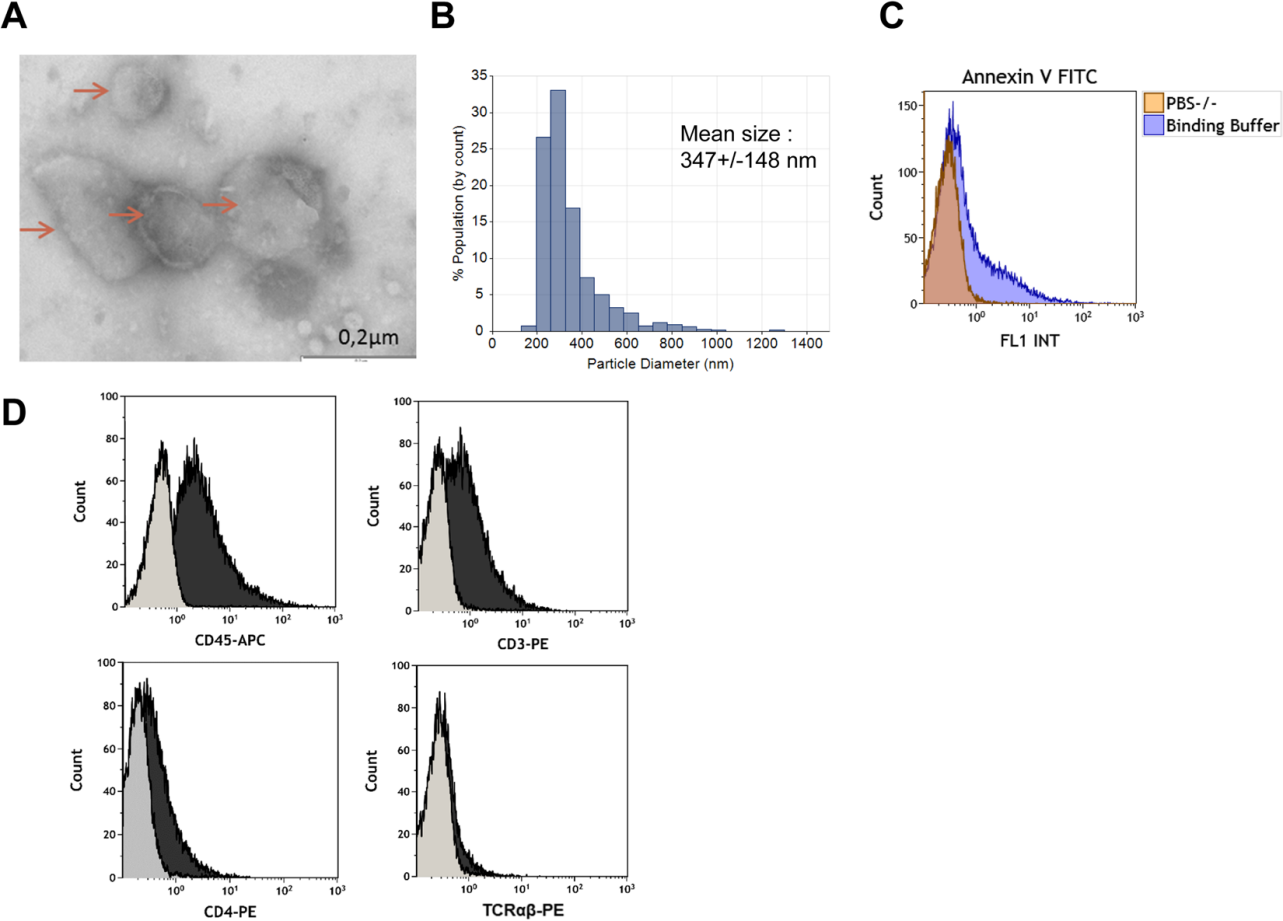
Characterization of CD4 T cell-derived EVs from ART-naive HIV-1-infected patients. (**A**) Transmission electron micrograph of CD4 T cell-derived EVs. Vesicles displaying sizes corresponding to EVs are indicated by arrows. (**B**) Size distribution of CD4 T cell-derived EVs measured by TRPS. Data are expressed as the mean size +/− SEM. (**C**) Representative histograms of PS expression detected by Annexin V labeling. Control experiments were performed in the presence of Ca^2+^ free PBS (orange) instead of Ca^2+^ containing buffer (blue). (**D**) Representative flow cytometry histograms of isolated Annexin V+ CD4 T cell-derived EVs stained with APC-tagged anti-CD45, PE-tagged anti-CD3, PE-tagged anti-CD4, and PE-tagged anti-TCRαβ (black). The control experiments stained with appropriate isotype control antibodies are displayed in grey.

To test the hypothesis that HIV-1 infection can induce miRNA expression in CD4 T cells, which may be transferred to endothelial cells by EVs, we first developed an *ex vivo* model to obtain CD4 T cell-derived EVs from HIV-1-infected patients. The clinical and biological characteristics of the patients are summarized in Table 1. Using a combination of transmission electron microscopy (TEM), tunable resistive pulse sensing (TRPS) and flow cytometry, we characterized the EVs shed *in vitro* by CD4 T cells from HIV-1-infected patients. TEM performed on CD4 T cell-derived EVs revealed that the CD4 T cells secrete vesicles displaying a characteristic shape of EVs (Fig. 1A). In addition, the size distribution of EVs was confirmed using TRPS, demonstrating that CD4 T cell-derived EVs have a mean size of 347 +/− 148 nm (Fig. 1B). As expected, flow cytometry showed that CD4 T cell-derived EVs were also positive for PS detected by Annexin V labeling (Fig. 1C). In addition, CD4 T cell-derived EVs were strongly positive for CD45, positive for CD3 and weakly positive for CD4 and TCRαβ (Fig. 1D). Flow cytometry showed the absence of EVs derived from endothelial cells, platelets, myeloid cells or erythrocytes (Supplemental Fig. 1A), as well as the absence of apoptotic bodies (Supplemental Fig. 1B). The absence of HIV-1 virus was determined via the detection of HIV-1 RNA using reverse transcriptase polymerase chain reaction (RT-PCR) (data not shown). In conclusion, vesicle preparations obtained from circulating CD4 T cells correspond to the morphological and phenotypic definition of CD4 T cell-derived EVs.

**Table 1:** Clinical characteristics of the study subjects.

	Healthy subjects	ART-naive HIV-1-infected patients	p
Number	5	5	—
Men, n (%)	5 (100%)	5 (100%)	—
Age (years)	41.4 ± 5,5*	41.4 +/− 5.9*	NS
Plasmatic HIV-1 viral load (copies/ml)	N/A	4839.8 +/− 3311.6*	—
Circulating CD4 T cell (/mm^3^)	796 +/− 230*	622 +/− 183*	NS
Years from diagnosis	N/A	4.1 +/− 1.3*	—
HCV Co-infection	0/5	0/5	—
HBC Co-infection	0/5	0/5	—
Nadir CD4 (/mm^3^)	N/A	585.6 +/− 167.8*	—

HIV-1-infected patients were naive for antiretroviral therapy (ART), which was a pre-requisite for recruitment. There were no differences between the groups regarding age (p = 0.790) and CD4 T count (p = 0.790). At inclusion, HIV-1-infected patients had a mean age of 41.4 ± 5.9 years and a mean CD4 T count of 622 ± 183 cells/mm^3^. At inclusion, healthy donors had a mean age of 41.4 ± 5.5 years and a mean CD4 T count of 796 ± 230 cells/mm^3^. HIV-1 infected patients had a documented infection history of 4.1 ± 1.3 years, a nadir CD4 count of 585 ± 167 cells/mm^3^ and a mean plasma viral load of 4.839 ± 3311 copies/mL in the absence of ART. All subjects were seronegative for both hepatitis B (HBV) and hepatitis C (HCV).

*Mean +/− SD; N/A: Not applicable; NS: Not significant; HCV hepatitis virus: HBV hepatitis B virus.

### miR-146b-5p is upregulated in CD4 T cells, CD4 T cell-derived EVs and circulating EVs from ART-naive HIV-1-infected patients

**Figure 2:**
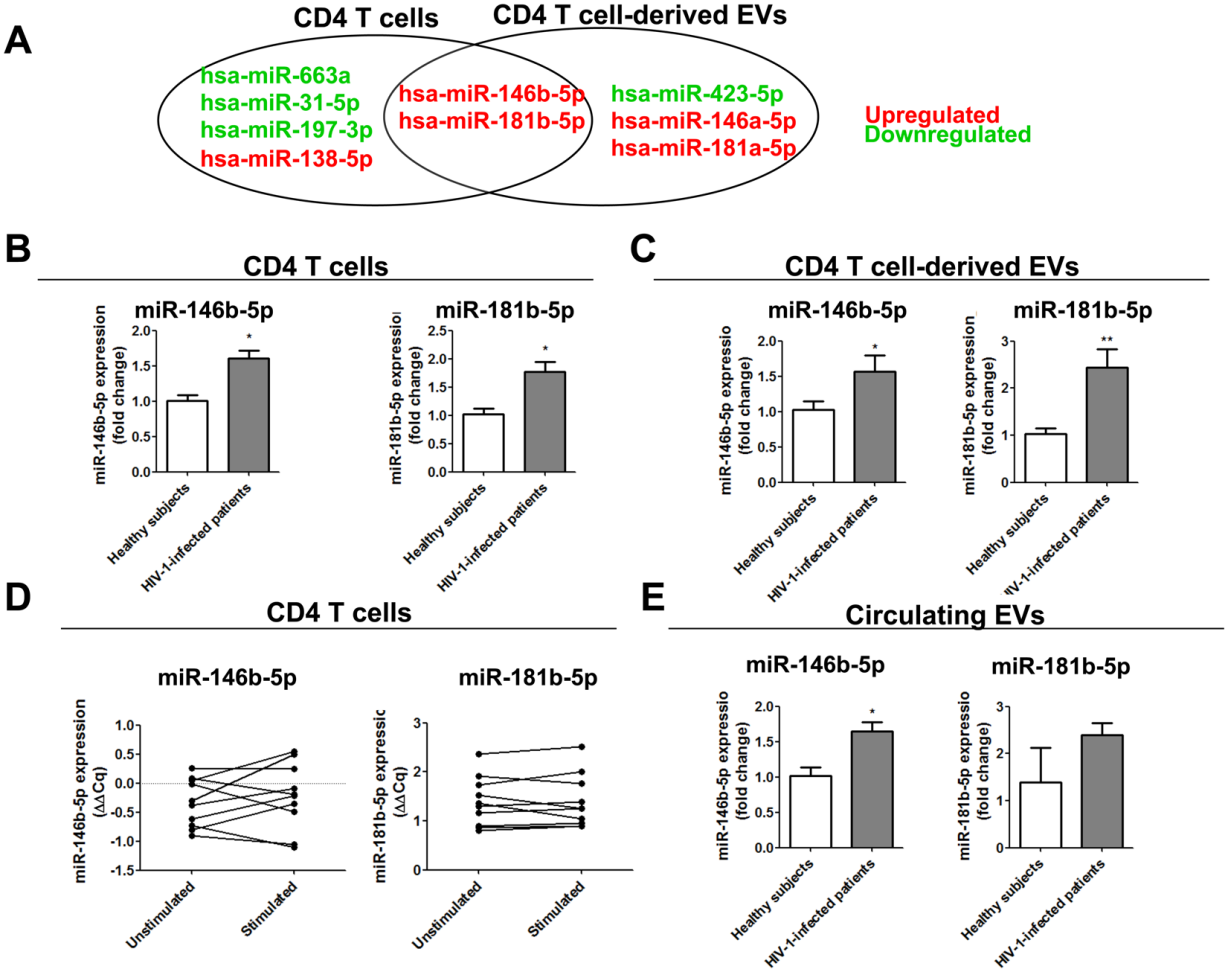
miR-146b-5p is upregulated in CD4 T cells, CD4 T cell-derived EVs and circulating EVs from ART-naive HIV-1-infected patients. (**A**) Venn diagram of the overlap of miRNA profiles in CD4 T cells and in CD4 T cell-derived EVs from ART-naive HIV-1-infected patients compared to those from healthy subjects. The differentially expressed miRNAs in CD4 T cell-derived EVs and circulating CD4 T cells are depicted in the form of two overlapping circles. miR-146b-5p and miR-181b-5p expression (fold change) in CD4 T cells (**B**) and CD4 T cell-derived EVs (**C**) from ART-naive HIV-1-infected patients compared those from healthy subjects. (**D**) Comparison of miR146b-5p and miR-181b-5p expression (ΔΔCq) in unstimulated or PAF/PMA-stimulated CD4 T cells from each study subject. (**E**) miR-146b-5p and miR-181b-5p expression (fold change) in circulating EVs from ART-naive HIV-1-infected patients compared to those from healthy subjects. Mean +/− SEM, *P < 0.05, **P < 0.01.

To identify miRNAs of interest in CD4 T cell-derived EVs from ART-naive HIV-1-infected patients, we evaluated the differentially expressed miRNAs in both CD4 T cells and CD4 T cell-derived EVs from ART-naive HIV-1-infected patients in comparison with CD4 T cells and CD4 T cell-derived EVs from healthy subjects thanks to a miRNome analysis (Fig. 2A). Among the 375 miRNAs analyzed in CD4 T cells, 6 miRNAs were differentially expressed in ART-naive HIV-1-infected patients: 3 (miR-146b-5p, hsa-miR-138-5p and miR-181b-5p) were upregulated, and 3 (hsa-miR-663a, hsa-miR-31-5p, and hsa-miR-197-3p) were downregulated. In CD4 T cell-derived EVs, 5 miRNAs were differentially expressed in ART-naive HIV-1-infected patients: 4 (miR-146a-5p, miR-146b-5p, miR-181a-5p and miR-181b-5p) were upregulated, and 1 (miR-423-3p) was downregulated. Interestingly, miR-146b-5p and miR-181b-5p were significantly upregulated in both CD4 T cells (Fig. 2B) and CD4 T cell-derived EVs (Fig. 2C) from HIV-1-infected patients. To verify that the upregulation of miRNAs observed in CD4 T cell-derived EVs was not induced by platelet-activating factor (PAF)/phorbol 12-myristate 13-acetate (PMA) stimulation, we compared the expression levels of both miR-146b-5p and miR-181b-5p between unstimulated and PAF/PMA-stimulated CD4 T cells from each subject. No significant difference was found for either miRNA (Fig. 2D). Finally, we compared the expression of both miRNAs in circulating EVs purified from the plasma of ART-naive HIV-1-infected patients and healthy subjects. While a significant increase was detected for miR-146b-5p in circulating EVs from ART-naive HIV-1-infected patients, no significant difference was found for miR-181b-5p (Fig. 2E). All these data demonstrate that miR-146b-5p and miR-181b-5p are overexpressed in CD4 T cells of ART-naive HIV-1-infected patients and that the overexpression was also found in EVs generated from these cells independently of PAF/PMA stimulation. Based on the difference in expression detected in plasma EVs, we chose to focus on miR-146b-5p for the next *in vitro* and *in vivo* studies.

### CEM-EVs protect miR-146b-5p from RNase degradation

**Figure 3:**
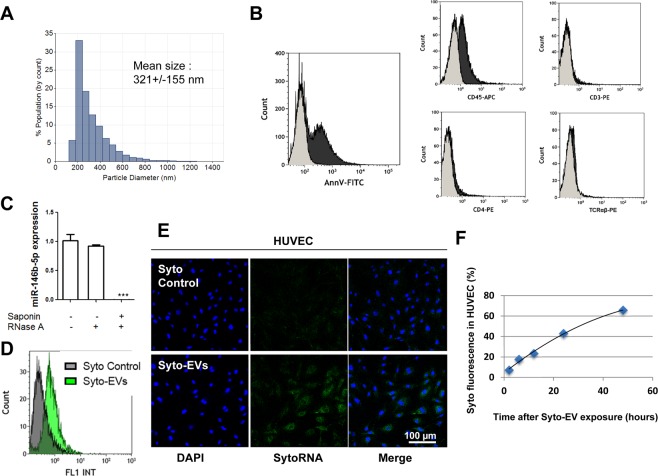
CEM-EVs protect their miRNA cargo from RNase degradation and transfer RNAs to HUVEC. (**A**) Size distribution of CEM-EVs analyzed by TRPS. Data are expressed as the mean size +/− SEM. (**B**) Representative flow cytometry histograms of isolated CEM-EVs stained with FITC-conjugated Annexin V, APC-tagged anti-CD45, PE-tagged anti-CD3, PE-tagged anti-CD4 and PE-tagged anti-TCRαβ (black). For Annexin V labeling, negative control experiment was performed in Ca^2+^ free PBS (grey). For other experiments, appropriate isotype antibodies was used as negative control (grey). (**C**) Prior to RNA extraction, intact CEM-EVs were either left untreated or treated with RNase A alone or with both saponin and RNase A. Samples were then subjected to RNA extraction, and miR-146b-5p expression was determined by quantitative RT-PCR; ***P < 0.001, n = 3. (**D**) Flow cytometry of HUVEC incubated for 2 hours with Syto control (gray curve) or with Syto-EVs (green curve) Data are representative of 3 different experiments. (**E**) Confocal microscopy of HUVEC incubated for 2 hours with Syto control or with Syto-EVs. HUVEC nuclei were labeled with DAPI (blue). (**F**) Time-course curve of RNA transfer by determining fluorescent intensity of HUVEC by flow cytometry at specific times (2, 6, 12, 24, 48 hours). Data are representative of 3 different experiments.

To investigate the ability of EVs to protect miRNA content from degradation by RNase, we used EVs generated from CEM cells (CEM-EVs). We confirmed that CEM-EVs displayed a mean size of 321 +/− 155 nm (Fig. 3A). As assessed by flow cytometry, CEM-EVs were positive for PS detected by Annexin V labeling, for CD45, weakly positive for CD4, negative for CD3 and TCRαβ (Fig. 3B). CEM-EVs were treated or not with RNase A alone or both saponin and RNase A prior to RNA extraction. miR-146b-5p expression level was further determined by quantitative RT-PCR (Fig. 3C). Compared to the basal miR-146b-5p expression level, RNase A treatment did not significantly reduce miR-146b-5p expression level. When RNase A treatment was preceded by saponin permeabilization, miR-146b-5p expression level was dramatically reduced, indicating that EV permeabilization was required for the RNase A-mediated degradation of miRNA content within EVs. Thus, these data show that miR-146b-5p located inside EVs is protected from RNase degradation.

### CEM-EVs transfer RNAs to endothelial cells *in vitro*

We next generated an *in vitro* model system using CEM cells and human umbilical vein endothelial cells (HUVEC) to determine whether CEM-EVs can transfer RNAs to endothelial cells. CEM-EVs stained with SytoRNA (Syto-EVs), a green fluorescent cell dye selective for RNA, were incubated on a monolayer of HUVEC for 48 hours at 37 °C. To exclude the presence of extravesicular dye in EVs, the samples were subjected to size exclusion chromatography (SEC). As a control for purification, HUVEC were incubated with an equivalent amount of dye alone previously subjected to SEC (called Syto Control). Flow cytometry showed an increase in fluorescence in HUVEC after Syto-EV exposure for 2 hours (Fig. 3D), which was confirmed by confocal microscopy (Fig. 3E). Flow cytometry also demonstrated a time-dependent increase in fluorescence in HUVEC after Syto-EV exposure with a maximum at 48 hours (Fig. 3F). Altogether, these results demonstrate that CEM-EVs are able to transfer RNAs to endothelial cells after incubation with EVs.

### CEM-EVs transfer miR-146b-5p mimics to endothelial cells *in vitro* in a PS-dependent manner

**Figure 4:**
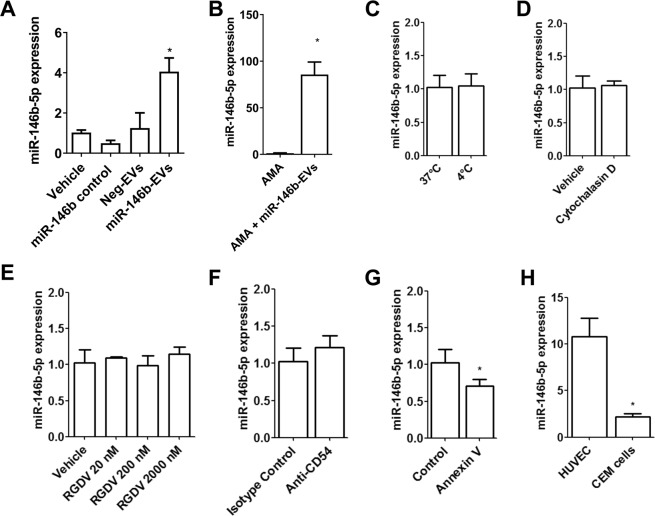
miR-146b-EVs transfer miR-146b-5p to HUVEC *in vitro* in a PS-dependent manner. (**A**) HUVEC were incubated with miR-146b-EVs at 37 °C for 24 hours. miR-146-5p expression level was determined by quantitative RT-PCR. Cells incubated with vehicle, miR-146b control and Neg-EVs were used as controls. Data are normalized to U6 snRNA expression. Mean +/− SEM, *P < 0.05, n = 3. (**B**) HUVEC were incubated with 50 µg/L α-amanitin at 37 °C for 8 hours prior to the addition of miR-146b-EVs at 37 °C for 24 hours. miR-146-5p expression level was determined by quantitative RT-PCR. Cells incubated with α-amanitin alone were used as controls. Data are normalized to U6 snRNA expression. Mean +/− SEM, *P < 0.05, n = 3. (**C**) to (**G**) Effects of potential inhibitors on miR-146b-5p transfer by miR-146b-EVs. Under both conditions, HUVEC were incubated with miR-146b-EVs at 37 °C for 20 hours (and 4 °C for 2 hours in **C**). HUVEC were pretreated with 10 µg/mL cytochalasin D (**D**), 20–2,000 nmol/L RGDV peptide (**E**) or 1 µg/mL anti-CD54 blocking antibody (**F**) for 1 hour prior to the addition of miR-146b-EVs for 20 hours. (**G**) miR-146b-EVs were preincubated for 15 minutes with 1 µM Annexin V before being added to HUVEC. (**H**) HUVEC or CEM cells were incubated with miR-146b-EVs at 37 °C for 20 hours. (**D**) to (**F**) miR-146-5p expression level was determined by quantitative RT-PCR. Cells incubated with vehicle (or isotype control in **F**) were used as controls. Mean +/− SEM, *P < 0.05, n = 3 to 4. AMA: α-amanitin.

We next investigated the capacity of CEM-EVs to transfer miR-146b-5p into endothelial cells. To this end, we investigated whether exogenous miRNAs can be transferred into HUVEC using EVs generated by CEM cells transfected with miR-146b-5p mimics. To verify the efficient packaging of miRNAs into EVs using this method, we first generated EVs from CEM cells transfected with hiv1-miR-TAR-5p mimics (TAR-EVs). hiv1-miR-TAR-5p is an HIV-1-specific miRNA that is not expressed in uninfected human cells^15,16^. TAR-EV samples were treated with RNase A alone or with both saponin and RNase A prior to SEC and RNA extraction. The expression of hiv1-miR-TAR-5p was detected in CEM-EVs after RNase A treatment, but it was susceptible to RNase A digestion after the disruption of the EV membrane with saponin (Supplemental Fig. 2A). This indicates that hiv1-miR-TAR-5p is efficiently packaged inside EVs. To eliminate extravesicular miRNAs, both EV samples were systematically treated with RNase A prior to SEC. Quantitative RT-PCR analysis showed that hiv1-miR-TAR-5p was present in HUVEC incubated for 48 hours with TAR-EVs (Supplemental Fig. 2B). As a control of purification, HUVEC were incubated with an equivalent amount of hiv1-miR-TAR-5p mimics alone and subjected to RNase A treatment prior to SEC (called TAR control). In this condition, we did not detect hiv1-miR-TAR-5p expression in HUVEC. These results indicate that CEM-EVs are able to transfer exogenous miRNAs into endothelial cells. In the same way, we generated EVs from CEM cells transfected with miR-146b-5p mimics (miR-146b-EVs) or negative control mimics (Neg-EVs). We next exposed identical concentrations of miR-146b-EVs or Neg-EVs to HUVEC. Quantitative RT-PCR analysis showed that miR-146b-5p expression was significantly increased in HUVEC incubated for 48 hours with miR-146b-EVs compared to that in HUVEC incubated with vehicle or with Neg-EVs (Fig. 4A). As a control, HUVEC were incubated with equivalent amounts of miR-146b-5p mimics alone and subjected to RNase A treatment prior to SEC (called miR-146b control), and we did not observe any differences compared to HUVEC exposed to vehicle treatment in terms of miR-146b-5p expression. To confirm that miR-146b-5p is transferred from CEM-EVs to endothelial cells and not transcriptionally induced, we incubated HUVEC with miR-146b-EVs in the presence of α-amanitin, an inhibitor of transcriptional activation^17^, or with α-amanitin alone for 8 hours. A 20-hour incubation of miR-146b-EVs significantly increased miR-146b-5p expression in HUVEC in the presence of α-amanitin (Fig. 4B), indicating the effective transfer of miR-146b-5p mimics into HUVEC.

We next investigated the mechanisms involved in the transfer of miR-146b-5p to endothelial cells via miR-146b-EVs. Under our experimental conditions, miR-146b-5p transfer was not reduced when HUVEC were incubated at 4 °C (Fig. 4C), pretreated with cytochalasin D for 8 hours in order to inhibit actin polymerization (Fig. 4D), or treated with RGDV peptide (Fig. 4E) or anti-ICAM-1 blocking antibody (anti-CD54) (Fig. 4F) for 1 hour to inhibit attachment via integrins. However, miR-146b-5p transfer was significantly reduced by approximately 30% when CEM-EVs were pretreated with blocking Annexin V for 15 minutes (Fig. 4G), suggesting a PS-dependent mechanism. In addition, miR-146b-5p transfer was significantly reduced when the HUVEC were replaced with CEM cells in terms of recipient cells (Fig. 4H), suggesting that endothelial cells were preferentially targeted by EVs. Altogether, these data demonstrate that miR-146b-5p mimics can be transferred to endothelial cells by miR-146b-EVs in a PS-dependent manner.

### miR-146b-EVs decrease endothelial activation *in vitro* via the downregulation of IRAK1, TRAF6 and ICAM-1/VCAM-1 expression

**Figure 5:**
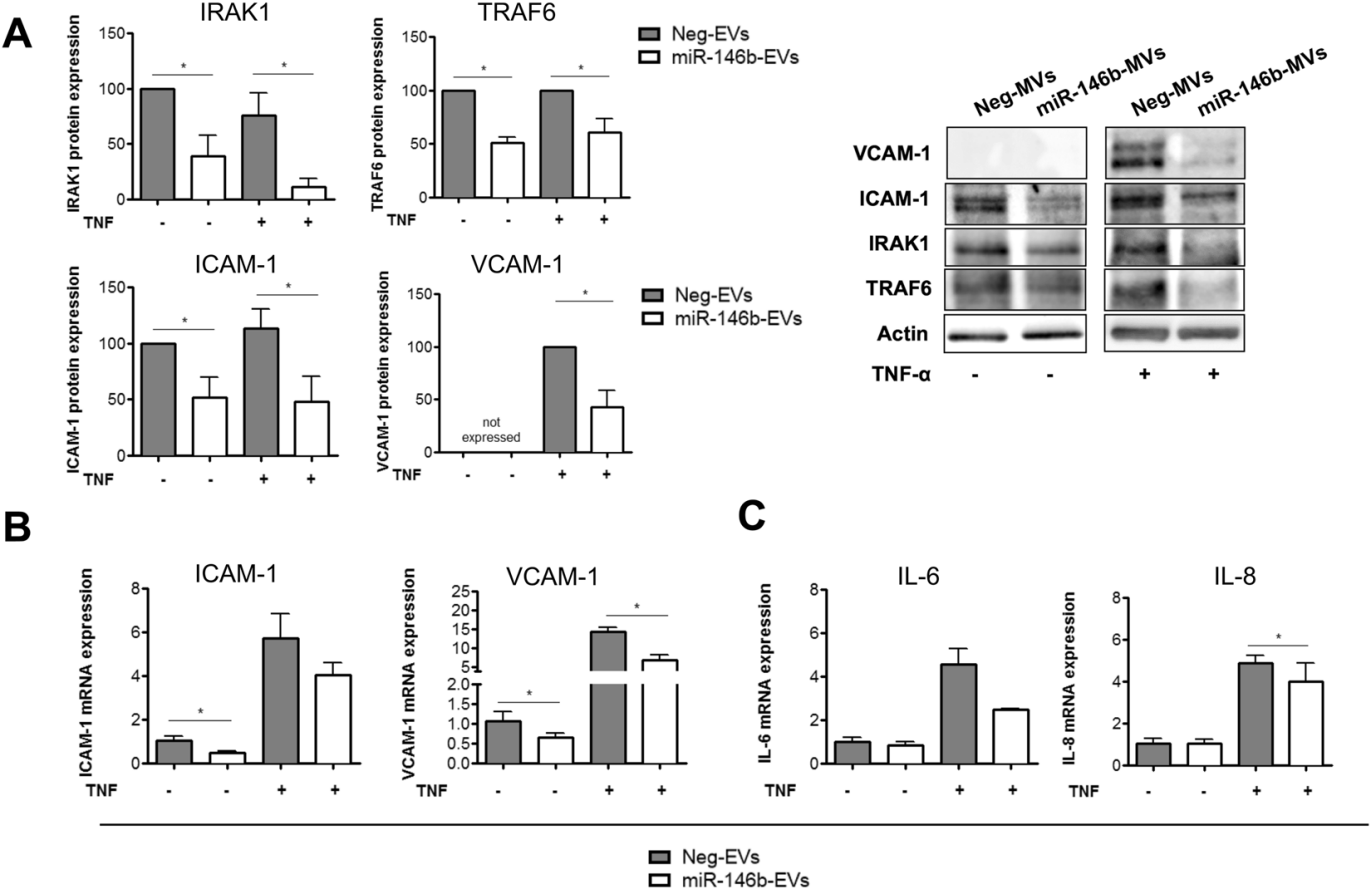
miR-146b-EVs repress endothelial activation *in vitro* via the downregulation of IRAK1 and TRAF6 expression. (**A**) HUVEC were incubated with miR-146b-EVs or Neg-EVs at 37 °C for 48 hours prior to the addition of TNFα or vehicle for 4 hours. (**A**) Western blot analysis of IRAK1, TRAF6, ICAM-1 and VCAM-1 protein expression in HUVEC. Blots come from different parts of the same gel as shown by delineation. After the quantification of protein expression, data were normalized to actin expression and presented relative to the data in unstimulated HUVEC incubated with Neg-EVs. Mean +/− SEM, *P < 0.05, n = 3 to 4. (**B**,**C**) HUVEC were incubated with miR-146b-EVs or Neg-EVs at 37 °C for 24 hours prior to the addition of TNFα or vehicle for 4 hours. Quantitative RT-PCR analysis of *ICAM-1*, *VCAM-1* (**B**), *IL-6* and *IL-8* (**C**) mRNA expression in HUVEC. Data are normalized to *GAPDH* expression and presented relative to unstimulated HUVEC incubated with Neg-EVs. Mean +/− SEM, *P < 0.05, n = 3.

Next, we sought to determine the *in vitro* effects of miR-146b-5p transfer on endothelial cells. For this purpose, miR-146b-EVs were incubated with unstimulated and tumor necrosis factor α(TNFα)-stimulated HUVEC. As a negative control, Neg-EVs were incubated with these cells. A 48-hour incubation of miR-146b-EVs decreased the expression of miR-146b-5p targets, such as TRAF6 and IRAK1, in unstimulated and TNFα-stimulated HUVEC as assessed by western blot analyses (Fig. 5A). Next, we assessed the effect of miR-146b-5p transfer on the expression of endothelial adhesion molecules. At 4 hours after TNFα incubation, *ICAM-1* and *VCAM-1* mRNA expression were found to be induced by approximately 6-fold and 15-fold, respectively, in HUVEC incubated with Neg-EVs (Fig. 5B). Interestingly, miR-146b-EVs significantly reduced ICAM-1 protein expression in both unstimulated and TNFα-stimulated HUVEC and VCAM-1 protein expression in TNFα-stimulated HUVEC (Fig. 5A). Additionally, a 24-hour incubation with miR-146b-EVs significantly reduced the mRNA expression of *ICAM-1* in unstimulated HUVEC and significantly reduced the mRNA expression of *VCAM-1* in both unstimulated and TNFα-stimulated HUVEC (Fig. 5B). In addition, we assessed the impact of miR-146b-5p transfer on the expression of pro-inflammatory genes such as interleukin (IL)-6 and IL-8. We found that miR-146b-EVs significantly reduced the mRNA expression of *IL-8* in TNFα-stimulated HUVEC (Fig. 5C). There was also a trend toward a decrease in *IL-6* mRNA expression in TNFα-stimulated HUVEC (Fig. 5C).

### miR-146b-EVs transfer miR-146b-5p mimics to lungs *in vivo*

**Figure 6:**
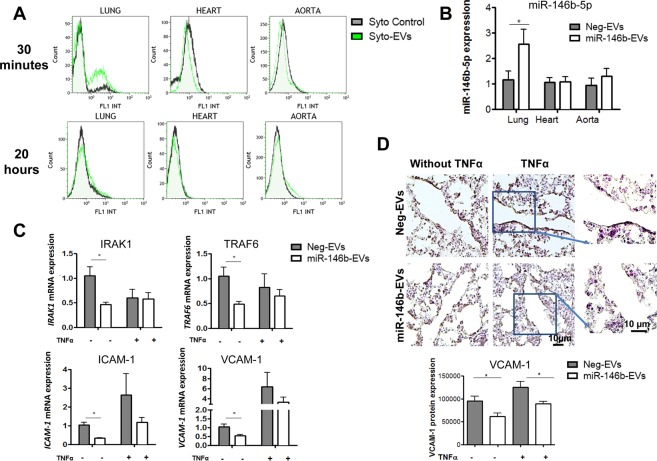
miR-146b-EVs transfer miR-146b-5p mimics to lungs *in vivo* and repress endothelial activation *in vivo*. (**A**) Flow cytometric analyses of lungs, heart and aorta from mice injected with Syto control (gray curve) or with Syto-EVs (green curve) after 30 minutes or 20 hours. Data are representative of 3 different experiments. (**B)** Mice were injected with miR-146b-EVs or Neg-EVs for 24 hours. miR-146-5p expression level in lungs, heart and aorta was determined by quantitative RT-PCR. Data are normalized to U6 snRNA expression and compared to the values in each organ from mice injected with Neg-EVs. Mean +/− SEM, *P < 0.05, n = 4 to 5. (**C**,**D**) Mice were injected with miR-146b-EVs or Neg-EVs 24 hours prior to the injection of vehicle or TNFα for 4 hours. Data are presented relative to values in vehicle injected-mice treated with Neg-EVs. Mean +/− SEM, *P < 0.05, n = 4 to 5. (**C**) Quantitative RT-PCR analysis of *IRAK1*, *TRAF6*, *ICAM-1* and *VCAM-1* mRNA expression in lungs. Data are normalized to *GAPDH* expression. (**D**), Representative micrographs of lungs with VCAM-1 staining, and quantitative analysis of VCAM-1 staining.

Next, we investigated whether CEM-EVs can transfer RNAs *in vivo*. Syto-EVs were intravenously (i.v.) injected into mice for 30 minutes or 20 hours. As a negative control, the mice were injected with Syto control. To evaluate the delivery of Syto-EVs after injection, lungs, heart, aorta (Fig. 6A), spleen, kidneys and liver (Supplemental Fig. 3) were analyzed by flow cytometry. Flow cytometry of Syto fluorescence demonstrated that CEM-EVs transfer RNAs to lung tissues within 30 minutes, as depicted by the presence of fluorescence signals, whereas no fluorescence was detected after 20 hours.

Next, by analyzing the expression of miR-146b-5p in the lungs, heart and aorta of mice, we investigated whether miR-146b-EVs can efficiently transfer miR-146b-5p mimics *in vivo*. Interestingly, the expression of miR-146b-5p in the lungs from mice injected with miR-146b-EVs was approximately 2.5-fold higher than that in the lungs from mice injected with Neg-EVs (Fig. 6B), whereas there were no changes in miR-146b-5p expression in the aorta or the heart (Fig. 6B), indicating that miR-146b-EVs can transfer miR-146b-5p mimics to lungs.

### miR-146b-EVs decrease endothelial activation in lungs *in vivo* via the downregulation of IRAK1, TRAF6 and ICAM-1/VCAM-1 expression

We next investigated whether the systemic administration of miR-146b-EVs can repress endothelial activation in lungs. To this end, miR-146b-EVs were injected into mice 24 hours prior to the injection of vehicle or TNFα for 4 hours. As a negative control, Neg-EVs were injected. miR-146b-EV injection decreased the mRNA expression of miR-146b-5p targets, such as *TRAF6* and *IRAK1*, as assessed by quantitative RT-PCR (Fig. 6C). At 4 hours after TNFα injection, the expression of *ICAM-1* and *VCAM-1* mRNAs was found to be induced by approximately 3-fold and 6-fold, respectively, in the lung tissues of mice injected with Neg-EVs (Fig. 6C). Interestingly, the systemic administration of miR-146b-EVs significantly reduced the expression of *ICAM-1* and *VCAM-1* mRNAs in the lungs of vehicle-injected mice (Fig. 6C). There was also a trend toward a decrease in *ICAM-1* and *VCAM-1* mRNA expression in TNFα-injected mice, but it did not reach significance (Fig. 6C). This could be explained by the fact that in this experiment, all cells comprising the lungs were analyzed and not the EV-targeted endothelial cells alone.

In addition, we studied VCAM-1 expression in lungs at the protein level. VCAM-1 protein expression was examined in lungs by immunohistochemical techniques. At 4 hours after TNFα injection, VCAM-1 protein expression was found to be induced by approximately 1.3-fold in lung sections from mice injected with Neg-EVs. The systemic administration of miR-146b-EVs significantly reduced VCAM-1 protein expression in the endothelium of lungs from vehicle- and TNFα-injected mice by 35% and 20% respectively (Fig. 6D). These findings demonstrate that miR-146b-EVs can deliver miR-146b-5p to lungs *in vivo* and thereby repress endothelial activation.

## Discussion

**Figure 7:**
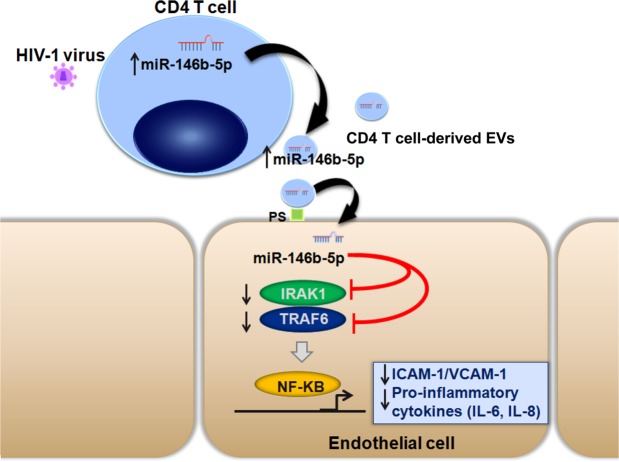
Pathophysiological hypothesis. Proposed model of how CD4 T cell-derived EVs act in vascular repair by counteracting endothelial activation in HIV-1 infection. Upon HIV-1 infection, the miRNA contents of CD4 T cells and CD4 T cell-derived EVs are altered, especially miR-146b-5p, which is upregulated. miR-146b-5p is incorporated into CD4 T cell-derived EVs and could be protected from RNase degradation in the bloodstream. CD4 T cell-derived EVs transfer miR-146b-5p to endothelial cells in a phosphatidylserine (PS)-dependent manner and lead to a decreased expression of nuclear factor-κB (NF-κB)-responsive molecules, including ICAM-1 and VCAM-1, through the downregulation of IRAK1 and TRAF6.

The mechanisms of endothelial dysfunction associated with HIV-1 infection are poorly understood. We show here that HIV-1 infection leads to the upregulation of miR-146b-5p in CD4 T cell-derived EVs from ART-naive HIV-1-infected patients. T cell-derived EVs overexpressing miR-146b-5p mimics protect their miRNA cargos from RNase degradation, transfer miR-146b-5p mimics to endothelial cells in a PS-dependent manner and reduce endothelial inflammatory responses *in vitro* and *in vivo*. This paracrine control of endothelial inflammatory responses mediated by EVs involves a reduction in the expression of the NF-κB-responsive molecules ICAM-1 and VCAM-1 through the downregulation of IRAK1 and TRAF6. Taken together, our results identify a new pathway involving a crosstalk between T cells and endothelial cells mediated by EV-transferred miR-146b-5p that represses endothelial activation (Fig. 7).

This study is the first to demonstrate that miR-146b-5p is upregulated in CD4 T cells, CD4 T cell-derived EVs and circulating EVs from ART-naive HIV-1-infected patients. Consistent with the chronic inflammatory status of HIV-1 patients, the expression of miR-146b has been reported to be induced by various inflammatory stimuli, such as lipopolysaccharide, TNFα, and IL-1β in different cell types^18–21^. Since their discovery in humans in 2006^18^, the role of miR-146b has been demonstrated in immune and inflammatory responses, autoimmunity, infectious context and cancer^22^. miR-146a and miR-146b have the ability to negatively regulate inflammation. The anti-inflammatory effects of the endogenous miR-146b have been described within different cell types, particularly in monocytes/macrophages^18,23^, dendritic cells^24^, T cells^25^, epithelial cells^19^ and endothelial cells^21^.

The role of EVs as vectors of miRNAs is a topic of growing interest in many diseases including inflammation, cancer and cardiovascular disorders^7,26,27^. The observation that miR-146 family members are passed from immune cells to other cell types via EVs to dampen inflammation has been reported by several groups^28–30^. Regarding HIV-1 infection, much effort has been focused on understanding the relationship between exosomes and HIV-1 infection^31^. Interestingly, we demonstrated here that the added values of packaging miR-146b-5p in CD4 T cell-derived EVs are to protect this miRNA from RNase degradation and to enable its passage through the bloodstream since miR-146b-5p was detected within circulating EVs isolated from the blood of HIV-1 infected patients. Our results are in agreement with a recent study, which showed that RNase A treatment of EV preparations prior to RNA isolation does not alter the RNA profile, thereby confirming the localization of RNAs within the EVs^26^.

Using EVs from the CEM T cell line overexpressing miR-146b-5p mimics incubated with HUVEC, we demonstrated the selective transfer of miR-146b-5p mimics into endothelial cells, through the specific overexpression of miR-146b-5p in endothelial cells and the subsequent demonstration of the functional impact of this transfer. In addition, the successful transfer of miR-146b-5p in the presence of α-amanitin, an inhibitor of RNA polymerase II, showed that the increase in the expression of miR-146b-5p in endothelial cells was not due to an immunomodulatory effect or transcriptional activation. Finally, we ensured that extravesicular miRNAs were absent in EV samples by combining RNase A pretreatment and SEC, because these miRNAs might independently influence the behavior of target cells. Different mechanisms responsible for EV internalization have been proposed, including clathrin-dependent endocytosis, caveolin-dependent endocytosis, phagocytosis, macropinocytosis and membrane fusion^32^. In our study, the data showed that the transfer of miR-146b-5p mimics did not involve endocytosis because it was neither inhibited by incubation at 4 °C nor suppressed by cytochalasin D. This transfer did not involve integrins or the adhesion molecule ICAM-1 expressed by the endothelium because the process was not inhibited by RDGV peptides or by the anti-ICAM-1 blocking antibody. We can hypothesize that miRNA could enter the cell through a membrane fusion mechanism that is independent of energy^33^. This would explain why incubation at 4 °C did not block the entry of miRNA into the cell. Interestingly, the miRNA transfer was reduced by the pretreatment of EVs with blocking Annexin V that binds PS. However, this miRNA transfer is not completely dependent on PS and could involve other EV surface molecules. Therefore, the predominant mechanism of this delivery need to be further explored.

Using the CEM model and HUVEC stimulated by TNFα, we demonstrated that miR-146b transferred by EVs decreases endothelial activation *in vitro* by targeting IRAK1 and TRAF6. Our results are in line with previous studies showing that IRAK1 and TRAF6 are the direct targets of the miR-146b^18,34^. Consequently, we observed the repression of inflammatory genes and proteins in the NF-κB pathway including ICAM-1, VCAM-1 but also IL-8, consistent with the inhibitory effects of miR-146b-5p in this pathway.

By injecting mice with miR-146b-EVs, we demonstrated the selective transfer of miR-146b-5p mimics by EVs *in vivo*. Indeed, EVs rapidly transfer RNAs to the lungs, within 30 minutes after i.v. injection. Although the *in vivo* uptake of exosomes has been previously reported, to the best of our knowledge, the *in vivo* transfer of miRNAs carried by T cell-derived EVs within the vascular compartment has never been demonstrated. Interestingly, we demonstrated that EVs transfer miR-146b-5p mimics, leading to the repression of target genes *IRAK1* and *TRAF6* 24 hours after injection. As a consequence, EVs repress endothelial inflammatory responses by decreasing the expression of the NF-κB-responsive molecules, ICAM-1 and VCAM-1 *in vivo*.

Interestingly, we showed that miR-146b transferred by EVs decreases endothelial inflammatory responses. Given their high circulating levels in many pathological conditions, especially in cardiovascular pathologies, EVs were initially considered as carriers of pro-coagulant and deleterious pro-inflammatory signals, thus amplifying and exacerbating the severity of pathologies^35^. More recent studies have revealed an alternative view, showing that EVs may also exert beneficial effects by protecting endothelial functions^36,37^. Outside the context of HIV-1 infection, previous studies have demonstrated the transfer of anti-inflammatory miRNAs to the endothelium by EVs. An earlier study described the transfer of miR-126 by apoptotic bodies of an endothelial origin triggering the production of CXCL2 thereby exerting a vasculoprotective effect *in vitro* and *in vivo*^38^. Another study showed that the transfer of the same miRNA, miR-126, by EVs of an endothelial origin could improve vascular regeneration *in vitro*^39^. The presence of anti-inflammatory paracrine regulations mediated by the transfer of anti-inflammatory miRNAs contained in exosomes of an endothelial origin has also been demonstrated in inflammation and septic shock^40^. To date, few studies have focused on the EV-mediated crosstalk between T cells and the endothelium. Several studies from Andriantsitohaina and colleagues have shown that EVs produced *in vitro* by activated and apoptotic T cells express the Sonic hedgehog morphogen and improve endothelial function^41–43^. Our study is the first to show the transfer of an anti-inflammatory miRNAs into the endothelium by CD4 T cell-derived EVs. Thus, this new mechanism likely models a protective response mediated by EVs, resulting from the crosstalk between CD4 T cells and the endothelium. We may speculate that, during HIV-1 infection, CD4 T cell-derived EVs overexpressing miR-146b-5p behave as carriers of vasculoprotective effectors that prevent endothelial dysfunction at the onset of viral infection. Indeed, the host could utilize the vesicle secretion machinery for its defense against viral infection. However, in the context of HIV-1 infection, whether this anti-inflammatory mechanism is beneficial or not remains to be elucidated.

Several limitations of our study warrant mention. Firstly, we used a cocktail of PAF/PMA to stimulate the EV release from CD4 T cells extracted from blood samples of HIV-1 patients and healthy subjects, which were used subsequently for miRNA profiling. We were aware that this stimulation could potentially induce changes in gene expression of these cells, but this protocol proved necessary to obtain sufficient material for the miRNome analysis. However, against this potential effect, we showed the absence of significant variations in expression of miR-146b-5p between stimulated and unstimulated cells for each subject whatever the HIV-1 statute. We cannot exclude there were induced changes in the miRNA loading into EVs, but the same stimulation protocol was used for patients and controls, thus reducing, in our opinion, the risk of bias when we compared the two conditions. Secondly, considering the same problem of limited available material from CD4 T cells and the very high quantity of EVs need for *in vitro* functional studies, we decided to use a model of T cell line (CEM) even if their gene expression patterns differ. This cell line allowed us to model, although not ideal, using a mimic transfection approach, the selective overexpression of miR-146b-5p identified in CD4 T cells of ART-naive HIV-1-infected. We considered that, whatever the genetic background of these cells, the effects of this overexpression could be evaluated compared to the miRNA control condition. Finally, we can question on the clinical relevance of our model. Our *in vitro* and *in vivo* results suggest a regulatory role of T cell-derived EVs overexpressing miR-146b-5p with a potential benefic impact on endothelial inflammation during HIV-1 infection. We could not faithfully control, in our model, the induced overexpression levels in EVs, which is probably higher compared to those in EVs released from CD4 T cells of patients. Furthermore, the quantity of EVs that we incubated on HUVECs *in vitro* or that we injected in mice is probably widely different from the *in vivo* context in patients. Concerning this last point, due to the limitation of samples, it was not possible, to date, to precisely characterize and quantify the rate of CD4 T cell-derived EVs circulating in patients’ blood and to evaluate indirectly the exposition level on their endothelium. We could not exclude that overexpression of miR-146b-5p quantified in circulating EVs can belong to various cell subtypes-derived EVs and not only to CD4 T cell-derived EVs. However, in contrary to our experimental protocol used, the exposition of endothelium in HIV-1 patients to CD4 T cell-derived EVs overexpressing miR-146b-5p, even if the level is probably lower, is chronic, which could potentially induce long-term effects.

In conclusion, we identified a new pathway involving a crosstalk between T cells and endothelial cells mediated by EV-transferred miR-146b-5p that represses endothelial activation. These data advance our understanding on chronic inflammatory responses affecting endothelial homeostasis, in infectious and non-infectious diseases and pave the way for potential new anti-inflammatory strategies.

## Materials and Methods

### Study subjects

Blood samples were obtained by venipuncture from 5 HIV-1-infected patients and 5 healthy subjects. The 5 HIV-1-infected patients were 40 +/− 10 years old with HIV-1 seropositivity for at least 2 years and a plasma viral load of >1000 copies/mL, and these patients had never received ART. The 5 healthy subjects were age-matched with the HIV-1-infected patients, and they displayed HIV-1 seronegativity on the day of inclusion. The characteristics of the subjects included in this study are summarized in Table 1. This study was approved by the CPP Sud Méditerranée I Ethics Committee (N° 2011-A000015-36), and informed consent was obtained from all subjects. All experiments within this study were performed in accordance with the Declaration of Helsinki.

### *Ex vivo* production model of CD4 T cell-derived EVs from subjects

The flowchart of the *ex vivo* production model of CD4 T cell-derived EVs is detailed in Supplemental Fig. 4. Circulating CD4 T cells were positively selected from blood samples by magnetic immunoseparation using a CD4 T cell isolation kit (Dynabeads Flowcomp™ Human CD4) according to the manufacturer’s protocol (Thermo Fisher Scientific, Courtaboeuf, France). CD4 T cells were processed for miRNA expression profiling or for EV production. EV release was induced upon the incubation of CD4 T cells in AIM-V medium (Thermo Fisher Scientific) with 500 nM PAF and 50 nM PMA (Calbiochem, San Diego, USA) for 20 hours. The culture media were collected and centrifuged for 5 minutes at 300 g to collect CD4 T cells which were processed for miRNA expression profiling. The supernatants were centrifuged for another 5 minutes at 2,500 g to remove cell debris. The supernatants were then concentrated using Vivaspin^®^ 20 units, 100 kDa MWCO Polyethersulfone (Sartorius, Dourdan, France). The cut off is 100 kDa, which retains the MVs and the exosomes in the concentrated medium^44,45^. The supernatants were subsequently filtered with 0.2 µm pores using the Ceveron MFU500 technology (Technoclone, Vienna, Austria) to isolate residues corresponding to EVs and to remove both exosomes and HIV-1 virus. CD4 T cell-derived EVs were recovered by resuspension of the 0.22 µm retentate material. EVs were washed two times and resuspended in phosphate-buffered saline (PBS) (Eurobio, Courtaboeuf, France). The presence of HIV-1 virus was determined via the detection of HIV-1 RNA using reverse transcriptase polymerase chain reaction (RT-PCR). We have submitted all relevant data of our experiments to the EV-TRACK knowledgebase (EV-TRACK ID: EV180070)^46^.

### Isolation of circulating EVs

Samples of circulating EVs were prepared from citrate-containing blood samples of the study subjects for miRNA profiling using quantitative RT-PCR. Platelet-poor plasma (PPP) was obtained by two centrifugation steps each at 2,500 g for 15 min. Subsequently, PPP was filtered with 0.2 µm pores using the Ceveron MFU500 technology (Technoclone, Vienna, Austria). Circulating EVs were recovered by resuspension of the 0.22 µm retentate material. EVs were washed two times and resuspended in PBS.

### Cells

The human lymphoid CEM T cell line was purchased from American Type Culture Collection and cultured in RPMI 1640 medium (Thermo Fisher Scientific) supplemented with 10% fetal bovine serum (Bioserum, Japan), 1% streptomycin, 1% penicillin and 1 mM pyruvate. HUVEC were isolated as previously reported^47^, grown in EGM™-2 (Lonza, Basel, Switzerland) and used between passages 3 and 4.

### Production and isolation of CEM cell-derived EVs overexpressing miRNA mimics

CEM cells were cultured for 48 hours to produce CEM-EVs. Supernatants were collected and centrifuged for 5 minutes at 300 g to remove cells and then centrifuged again for 5 minutes at 2,500 g to remove cell debris. Subsequently, supernatants were centrifuged for 90 minutes at 70,000 g at 4 °C and EV pellets were resuspended in PBS. In some experiments, EV samples were treated with 5 μg/ml of RNase A (Thermo Fisher Scientific) for 90 minutes at 37 °C. The reaction was stopped by adding 1 IU of RNaseOUT™ (Thermo Fisher Scientific) per 5 ng of RNase used. EV samples were then subjected to SEC using qEV original size exclusion columns (Izon, Cambridge, MA, USA). Then, 500 µl fractions were successively collected after the addition of EV samples to the top of the colums. Fractions 7 and 8 enriched in EVs were collected in final volume of 1 ml. When indicated, EV samples were treated with 20% saponin prior to RNase A treatment. To generate EVs from CEM cells overexpressing miRNA mimics, CEM-EVs were isolated from CEM cells transfected with miR-146b-5p mimics (called miR-146b-EVs), with hiv1-miR-TAR-5p mimics (called TAR-EVs) or with miRNA negative control (called Neg-EVs). The miRNA mimics miRVana™ hsa-miR-146b-5p (ID: MC10105), miRVana™ hiv1-miR-TAR-5p (ID: MC13575) and miRVana™ miRNA negative control were purchased from Thermo Fisher Scientific and used at a concentration of 25 nM. Transfection of CEM cells was performed using the magnetofection method (Oz Biosciences, Marseille, France) according to the manufacturer’s protocol. To check the efficiency of separation of EVs and extravesicular miRNA mimic by SEC, equivalent quantities of miR-146b-5p and hiv1-miR-TAR-5p mimics were subjected to RNase A treatment prior to SEC, and fractions 7 and 8 were collected and used as experimental controls (called “miR-146b control” and “TAR control”, respectively).

### Characterization of CD4 T cell-derived EVs and CEM-EVs

#### Transmission electron microscopy (TEM)

CD4 T cell-derived EVs fixed with 2% paraformaldehyde (PFA) in 0.1 M sodium cacodylate buffer (pH 7.4) were applied to 300 mesh formvar-grids for 20 minutes at room temperature (RT), washed two times in 0.1 M sodium cacodylate buffer (pH 7.4) and negatively stained with 0.3% phosphotungstic acid before observation with a JEOL JEM 1400 electron microscope (Peabody, USA). Images were captured with a CCD camera (Megaview III; SYS Olympus, Munich, Germany).

#### Tunable resistive pulse sensing (TRPS)

The size distribution of CD4 T cell-derived EVs and CEM-EVs was determined using the qNano instrument (Izon Science Ltd, Christchurch, New Zealand). The TRPS was previously described^48^. We followed manufacturers’ recommendations and NP400 nanopore membranes stretched between 43–47 nm were used. Voltage was set in 0.3–0.5 V to achieve a stable current 90–120 nA and the pressure at 0.8 kPa, with the root mean square noise below 10 pA. Calibration beads used were CPC200B and CPC400 (mean diameters 210 nm and 350 nm respectively) (all from Izon). Running electrolyte was PBS filtered under 0.1 µm. Measurement and analysis were performed with Izon Control Suite V3.3.3.2001 Software.

#### Flow cytometry

CD4 T cell-derived EVs and CEM-EVs were analyzed using high-sensitivity flow cytometry as previously described^49^. Thirty microliters of EV samples was incubated with the appropriate amount of specific antibodies and 10 μL of FITC-bound Annexin V reagent (Tau Technologies, Netherlands). Each stained sample was analyzed on a 3-laser Navios flow cytometer (Beckman-Coulter), based on a protocol standardized with Megamix-Plus FSC beads (BioCytex, Marseille, France). The following fluorescently-tagged antibodies were procured from Beckman Coulter: PE-tagged anti-TCRαβ, APC-tagged anti-CD45, PE-tagged anti-CD3, and PE-tagged anti-CD4 and their respective controls. The presence of EVs derived from different cellular origins was determined using PC7-tagged anti-CD41, APC-tagged anti-CD11b, Alexa 750 APC-tagged anti-CD235, PE-tagged anti-CD31 and their respective control antibodies. The presence of apoptotic bodies was determined by colabeling with FITC-bound Annexin V and DAPI (4′,6′-diamidino-2-phenylindole). Before analysis, CytoCount beads (Dako, Copenhagen, Denmark) were added as internal standards to samples to determine the concentration of EVs. Flow cytometry data were analyzed using Kaluza 1.5a Software (Beckman Coulter).

### miRNA profiling of CD4 T cell-derived EVs and CD4 T cells by quantitative RT-PCR

Total RNA was extracted using an miRNeasy mini kit (Qiagen, Courtaboeuf, France) according to the manufacturer’s instructions. RNA concentration was determined using a NanoDrop 1000 spectrophotometer (Thermo Fisher Scientific). Total RNA was then stored at −80 °C until use. miRNA expression profiling was performed by quantitative RT-PCR with a 384-well microRNA Human panel I (Exiqon) following the manufacturer’s recommendations. Briefly, 20 µl RNA were reverse transcribed in a 50 µl reaction volume using the miRCURY LNA^TM^ Universal RT miRNA PCR, Polyadenylation and cDNA synthesis kit (Exiqon). Subsequently, cDNA was 50x diluted and assayed in 10 µl volumes by PCR according to the manufacturer’s protocol. qPCR amplifications were performed using a LightCycler 480 Real-Time PCR System (Roche Diagnostics, Meylan, France). The determination of quantification cycle (Cq) and melting curve analysis was done using the Roche LC480 software and the 2^nd^ derivative method. Only molecules with a raw Cq ≤ 35 were considered to be expressed and included in subsequent data analyses. A first normalization was performed using interplate calibrators present in the 384-well microRNA Human panel I plate using the Exiqon GenEx qPCR analysis software. Normalization (ΔCq) was performed using the average of miRNAs expressed in all samples. Data were quantified using the ΔΔCq method. The change in miRNA expression was calculated as fold change relative to miRNA expression in the healthy control group using the 2^−ΔΔCq^ method. miRNAs were considered differentially expressed beyond a threshold of a 1.5-fold change with a p value ≤ 0.05.

### Quantification of individual miRNAs by quantitative RT-PCR

The expression level of specific miRNAs was determined using the miRCURY LNA™ Universal RT microRNA PCR system and ExiLENT SYBR^®^ Green master mix (Exiqon). Quantitative RT-PCR were performed in triplicate on a LightCycler 480 Real-Time PCR System (Roche). The expression of miR-146b-5p was normalized using the 2^−ΔΔCq^ method relative to the expression of miR-484 (hsa-miR-484 Product no.: 205636), which was defined by GeNorm and NormFinder softwares as the optimal reference (most stable) for CD4 T cells. Primers against the following targets were purchased from Exiqon: hsa-miR-146b-5p mimic (ID: 204553), hiv1-miR-TAR-5p mimic (ID: 204553) and hsa-miR-484 mimic (ID: 205636).

### *In vitro* EV uptake experiments

Syto RNA Select dyes (Molecular Probes) were used for RNA staining according to the manufacturer’s protocol to produce Syto RNA-stained CEM-EVs (hence called Syto-EVs) prior to SEC as described above. To check the absence of extravesicular dye, equivalent quantities of Syto RNASelect dyes were subjected to SEC in parallel. Fractions 7 and 8 were collected and used as experimental controls (called “Syto Control”). For flow cytometric analysis, HUVEC were seeded in 0.2% gelatin-coated (Sigma-Aldrich, St. Quentin Fallavier, France) 24-well plates at a concentration of 75,000-cells/mL for 24 hours to enable cell adhesion and then co-incubated with 2 × 10^6^ Syto-EVs for 2 hours, 6 hours, 12 hours, 24 hours and 48 hours. Then, the HUVEC were washed three times and harvested with trypsin. Cell suspensions were then analyzed by flow cytometry. The data were analyzed with the Kaluza 1.5a software after the target events were gated according to the forward and side scatter signals. HUVEC co-cultured with Syto-Control or without Syto-EVs served as controls. For confocal analysis, HUVEC were seeded in 0.2% gelatin-coated (Sigma-Aldrich) Lab-Tek II chamber slides (Thermo Fisher Scientific) at a concentration of 30,000 cells/mL and co-incubated with 1 × 10^6^ Syto-EVs for 2 hours. Then, the HUVEC were washed three times with PBS and fixed with 4% PFA (Thermo Fisher Scientific) for 10 min. DAPI staining of cellular nuclei was performed for 10 minutes at RT. Samples were analyzed using a Leica TCS SP5 II confocal microscope (Leica, Nanterre, France). In both experiments, HUVEC co-incubated with Syto control served as controls.

### *In vitro* effects of miR-146b-EVs on HUVEC

#### Quantitative RT-PCR

HUVEC were seeded in 0.2% gelatin-coated (Sigma-Aldrich) 24-well plates at a concentration of 75,000-cells/mL and co-incubated with 2 × 10^6^ TAR-EVs, miR-146b-EVs or Neg-EVs for 24 hours. When indicated, the HUVEC were stimulated using 10 ng/mL recombinant human TNFα (Miltenyi Biotech, Paris, France). Thereafter, the HUVEC were washed three times, and total RNA was extracted as described above. The expression level of hiv1-miR-TAR-5p or miR-146b-5p was determined as described above and was normalized to U6 snRNA expression (ID: 203907) (Exiqon). When indicated, HUVEC incubated with miR-146b control, TAR control or Neg-EVs were used as controls.

For mRNA quantification, RNA was reverse-transcribed to cDNA according to the manufacturer’s instructions using random hexamers (Thermo Fisher Scientific). cDNA from HUVEC was analyzed for the expression of *ICAM-1*, *VCAM-1*, *IL-6* and *IL-8* transcripts normalized to the expression of the housekeeping gene GAPDH using FastStart DNA Master PLUS SYBR Green I Reaction Mix on a Light Cycler 480 (Roche Diagnostics, Meylan, France). The sequences of oligonucleotide primers used for *IL-6*, *IL-8*, *ICAM-1*, *VCAM-1* and *GAPDH* amplification were as follows: IL-6 forward, 5′-AGCCCTGAGAAAGGAGACATGTAACAAG-3′; IL-6 reverse, 5′-TTCTGCAGGAACTGGATCAGGACTTT-3′; IL-8 forward, 5′-AGCCTTCCTGATTTCTGCAGCT-3′; IL-8 reverse, 5′-CCTTGGGGTCCAGACAGAGC-3′; ICAM-1 forward, 5′-GTGTACTGGACTCCAGAACGGG-3′; ICAM-1 reverse, 5′-ATGGTGATCTCTCCTCACCAGC-3′; VCAM-1 forward, 5′-TGCAAGTCTACATATCACCCAA-3′; VCAM-1 reverse, 5′-GTAGACCCTCGCTGGAACAG-3′; GAPDH forward, 5′-GGTGGTCTCCTCTGACTTCAACA-3′; and GAPDH reverse, 5′-GTTGCTGTAGCCAAATTCGTTGT-3′. HUVEC co-incubated with Neg-EVs served as controls.

To study the molecular mechanisms involved in the transfer of miR-146b-5p by CD4 T cell-derived EVs, HUVEC were incubated at 37 °C for 24 hours with 2 × 10^6^ miR-146b-EVs, at 4 °C in some experiments for 2 hours. Where indicated, HUVEC were preincubated at 37 °C with 50 µg/L α-amanitin (Sigma-Aldrich) for 8 hours or with 1 µg/mL anti-human CD54 (ICAM-1) blocking antibody (Clone HCD54) (Biolegend, San Diego, USA), 10 µg/mL cytochalasin D (Sigma-Aldrich) or 20–2000 nmol/L RGDV peptide (Polypeptide) for 1 hour. When indicated, miR-146b-EVs were preincubated for 15 minutes with 1 µM Annexin V (Sigma-Aldrich) before being added to HUVEC. For antibody blocking experiments, non-immune IgG was used as a negative control. In selected experiments, CEM cells were seeded in 0.2% gelatin-coated (Sigma-Aldrich) 24-well plates at a concentration of 75,000-cells/mL for 24 hours to enable semi-adhesion and then co-incubated with 2 × 10^6^ miR-146b-EVs. At the end of incubation with EVs, HUVEC were washed three times with PBS. miR-146b-5p transfer into recipient cells was detected via the quantification of miR-146b-5p by quantitative RT-PCR as described above.

#### Protein extraction and western blot analysis

HUVEC were seeded in 0.2% gelatin-coated (Sigma-Aldrich) 12-well plates at a concentration of 150,000 cells/mL, allowed to adhere for 24 hours and then co-incubated with 4 × 10^6^ miR-146b-EVs or Neg-EVs for 48 hours. HUVEC were washed three times and lysed in RIPA buffer supplemented with protease inhibitor cocktail. The lysates were centrifuged at 10,000 g for 10 min, and supernatants were collected. Protein concentrations were measured by a micro BCA protein assay kit (Thermo Fisher Scientific), and the remaining supernatants were stored at −80 °C until being tested by western blotting. Equal amounts of proteins (30–40 μg) were separated on 4–12% gradient SDS-polyacrylamide gels, blotted on cellulose C+ membranes and blocked in 5% BSA-TBS for 1 hour at RT. All primary antibody incubations were performed in blocking buffer overnight at 4 °C followed by incubation with horseradish peroxidase-conjugated anti-rabbit antibody for 1 hour at RT. Immuno-complexes were visualized by chemiluminescence using an ECL kit according to manufacturer’s instructions (Thermo Fisher Scientific). The following antibodies were used: rabbit anti-human polyclonal IRAK1 (4504, 1/1,000, Cell Signaling Technology), rabbit polyclonal TRAF6 (8028, 1/1,000, Cell Signaling Technology), rabbit polyclonal ICAM-1 (4915, 1/1,000, Cell Signaling Technology), rabbit polyclonal VCAM-1 (sc8304, 1/200, Santa Cruz Biotechnology), rabbit polyclonal β-actin (8457, 1/3,000, Cell Signaling Technology). HUVEC incubated with Neg-EVs were used as controls.

### Mice

Eight to 10-week-old male C57BL/6 mice were purchased from Janvier Labs. The mice were anesthetized with 3% sevoflurane in oxygen and intracardially perfused with PBS before being euthanized by cervical dislocation. All animal care and experiments were performed as recommended by the European Community Guidelines (directive 2010/63/UE) and approved by both the French Ministry of Higher Education, Research and Innovation and the Marseille Ethical Committee (approval APAFIS 1856-201509231032532).

### *In vivo* delivery of Syto-EVs

Each mouse (n = 3 per group) was injected with 500,000 Syto-EVs or Syto control by retro-orbital injection. The mice were sacrificed to harvest organs for flow cytometric analysis 30  minutes and 20 hours after the injection. Lungs, aorta, heart, liver, kidneys and spleen from each mouse were mechanically disrupted, and cell suspensions were analyzed by flow cytometry. Mice injected with Syto control were used as controls.

### *In vivo* delivery of miR-146b-EVs in a murine model of acute vascular inflammation

Each mouse (n = 4–5 per group) was i.v. injected with 500,000 miR-146b-EVs or Neg-EVs by retro-orbital injection. Recombinant mouse TNFα (2 μg/mouse) (Peprotech, Neuilly sur Seine, France) was intraperitoneally injected on the following day. Four hours after the TNFα injection, the mice were sacrificed to harvest organs for total RNA extraction and immunohistochemical analyses. Organs from mice injected with Neg-EVs were used as controls.

#### Quantitative RT-PCR

Lung, aorta and heart samples were mechanically disrupted and used for total RNA extraction. RNA was reverse transcribed to cDNA using random hexamers (Thermo Fisher Scientific) according to the manufacturer’s instructions. cDNA from lungs, aorta and hearts were analyzed for the expression of miR-146b-5p as described above, and miR-146b-5p levels were normalized to U6 snRNA levels (Product no: 203907) (Exiqon). cDNA from lungs was analyzed for expression of *IRAK-1*, *TRAF-6*, *ICAM-1* and *VCAM-1* transcripts using LightCycler 480 Probes Master (Roche Applied Science), and the corresponding gene expression was normalized to the expression of the housekeeping gene GAPDH. The following oligonucleotide primers were purchased from Thermo Fisher Scientific: *IRAK-1* (Mm01193538_m1), *TRAF-6* (Mm00493836_m1), *ICAM-1* (Mm00516023_m1), *VCAM-1* (Mm01320970_m1) and *GAPDH* (Mm99999915_g1).

#### Immunohistochemical analyses

Lungs were fixed in 4% PFA for 2 hours and placed in a 30% PBS sucrose solution overnight at 4 °C before being embedded in a cutting medium and frozen at −80 °C. Serial 8-µm transversal cryo-sections of lungs were obtained. VCAM-1 expression was analyzed using rabbit polyclonal anti-VCAM-1 antibody (sc-8304) from Santa Cruz Biotechnology. For detection, horseradish peroxidase (HRP)-conjugated goat anti-rabbit antibody was used (Thermo Fisher Scientific). Sections stained with secondary antibodies alone were used as controls of non-specific staining. The slides were examined under a light microscope. Lung sections were further counterstained with hematoxylin and eosin and examined at 20× or 63× magnification using a DMi8 advanced fluorescence microscope (Leica Microsystems, Germany). NIH ImageJ software version 1.45 was used for quantification.

### Statistical analysis

The data were expressed as the mean values ± s.e.m. of the indicated number of experiments. Statistical analyses were performed using the Prism software (GraphPad Software Inc., San Diego, CA, USA). Significant differences between two groups were determined using the Mann-Whitney test or unpaired Student’s t-test. Comparisons among related samples were performed using the Wilcoxon matched-pairs signed-rank test. A value of p ≤ 0.05 was considered significant.

## Supplementary information

Supplementary Information
