# Supplementary material for: Extracellular vesicles from T cells overexpress miR-146b-5p in HIV-1 infection and repress endothelial activation
Source: Sci Rep. 2019 Jul 16;9:10299. doi: 10.1038/s41598-019-44743-w (PMC6635508; doi:10.1038/s41598-019-44743-w)

Extracellular vesicles from T cells overexpress miR-146b-5p in HIV-1 infection and repress endothelial activation

Estelle Balducci[1,2], Aurélie S. Leroyer[1], Romaric Lacroix[1,2], Stéphane Robert[1], Dilyana Todorova[1], Stéphanie Simoncini[1], Luc Lyonnet[2], Corinne Chareyre[1], Olivia Zaegel-Faucher[3], Joëlle Micallef[4,5], Isabelle Poizot-Martin[3,6], Patrice Roll[7,8]* and Françoise Dignat-George[1,2]*

1. Aix Marseille Univ, INSERM, C2VN, Marseille, France
2. APHM, Hôpital La Conception, Laboratoire d'Hématologie, Marseille, France.
3. APHM, Hôpital Sainte-Marguerite, Service d'Immuno-hématologie clinique, Marseille, France
4. APHM, Hôpital la Timone, Service de Pharmacologie, Marseille, France
5. Aix Marseille Univ, CNRS, INT, Inst Neurosci Timone, Marseille, France
6. Aix Marseille Univ, Inserm U912 (SESSTIM), Marseille, France
7. Aix Marseille Univ, INSERM, GMGF, Marseille, France
8. APHM, Hôpital la Timone, Service de Biologie Cellulaire, Marseille, France.

*Both authors contributed equally to this work

Supplemental Figure 1

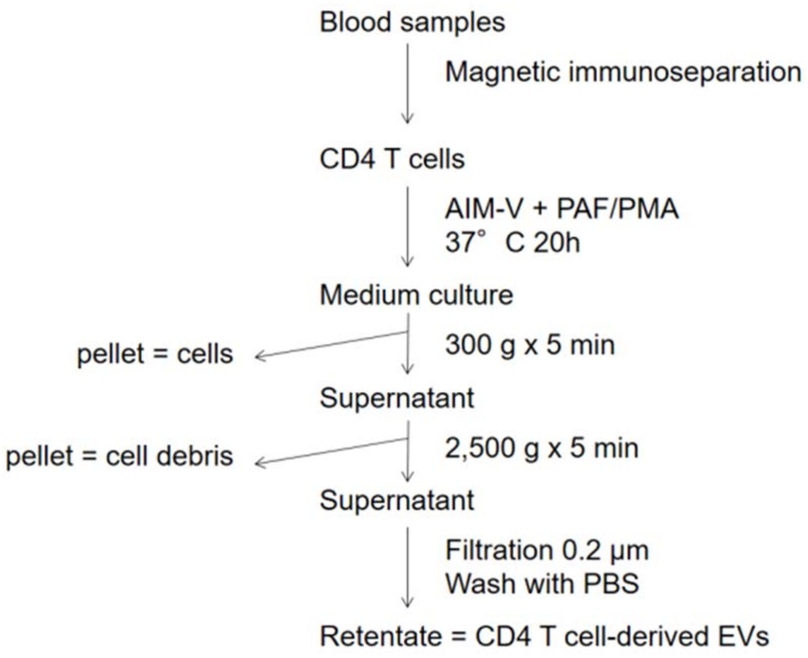

Supplemental Fig. 1. Flowchart of the *in vitro* production model of CD4 T cell-derived EVs from study subjects.

Supplemental Figure 2

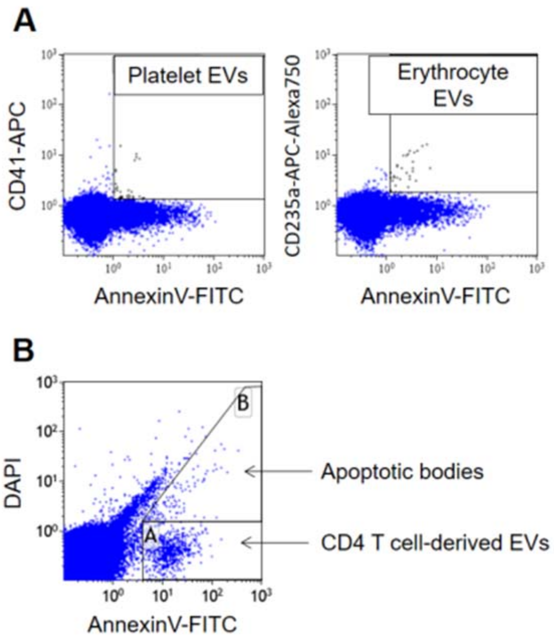

Supplemental Fig. 2. Characterization of CD4 T cell-derived EVs. A, Representative flow cytometry dot plots of CD4 T cell-derived EVs costained with fluorescein isothiocyanate (FITC)-conjugated Annexin V, PC7-tagged anti-CD41, APC-tagged anti-CD11b, Alexa 750 APC-tagged anti-CD235, the PE-tagged anti-CD31 antibodies. Platelet EVs are Annexin V+/CD41+, erythrocyte EVs are Annexin V+/CD235a+, myeloid EVs are Annexin V+/CD11b+, and endothelial EVs are Annexin V+/CD41-/CD31+. **B,** Representative flow cytometry dot plots of CD4 T cell-derived EVs costained with FITC-conjugated Annexin V and DAPI. EVs are Annexin V+/DAPI- (gate A), and apoptotic bodies are Annexin V+/DAPI+ (gate B).

Supplemental Figure 3

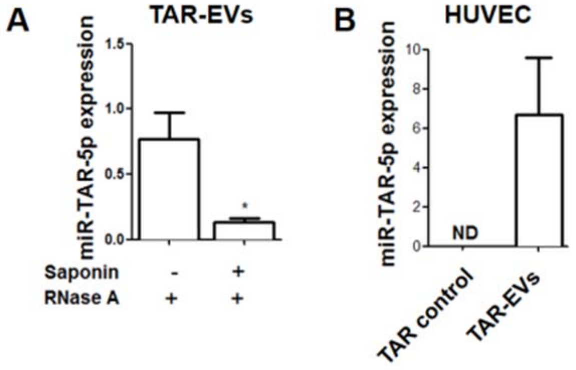

Supplemental Fig. 3. Validation of miRNA packaging in CEM-EVs. A, Prior to RNA extraction, intact TAR-EVs were treated with RNase A alone or with both saponin and RNase A. Samples were then subjected to RNA extraction, and hiv-1-miR-TAR-5p expression level was determined by quantitative RT-PCR; Mean +/- SEM, **P*<0.05, n=3. **B,** HUVEC were incubated with miR-146b-EVs or with Neg-EVs for 24 hours. The expression of hiv-1-miR-TAR-5p expression level in HUVEC was determined by quantitative RT-PCR. Data are normalized to U6 snRNA expression. For TAR control condition, hiv-1-miR-TAR-5p expression was set to 0 because hiv-1-miR-TAR-5p was not detected (ND) in HUVEC. Mean +/- SEM, **P*<0.05, n=3.

Supplemental Figure 4

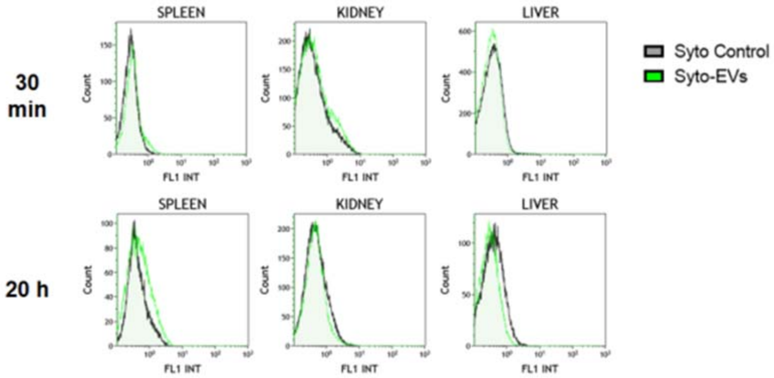

Supplemental Fig. 4. *In vivo* delivery of Syto-EVs in kidneys, liver and spleen. Flow cytometric analyses of kidneys, liver and spleen from mice injected with Syto control (gray curve) or Syto-EVs (green curve) after 30 minutes or 20 hours. Data are representative of 3 different experiments.

Supplemental Figure : Unprocessed original scans for all the blots in figure 5, only lanes 3 and 4 (without TNF) and 7 and 8 (with TNF) were shown in figure 5

VCAM-1 blot (left: without TNF, right: with TNF

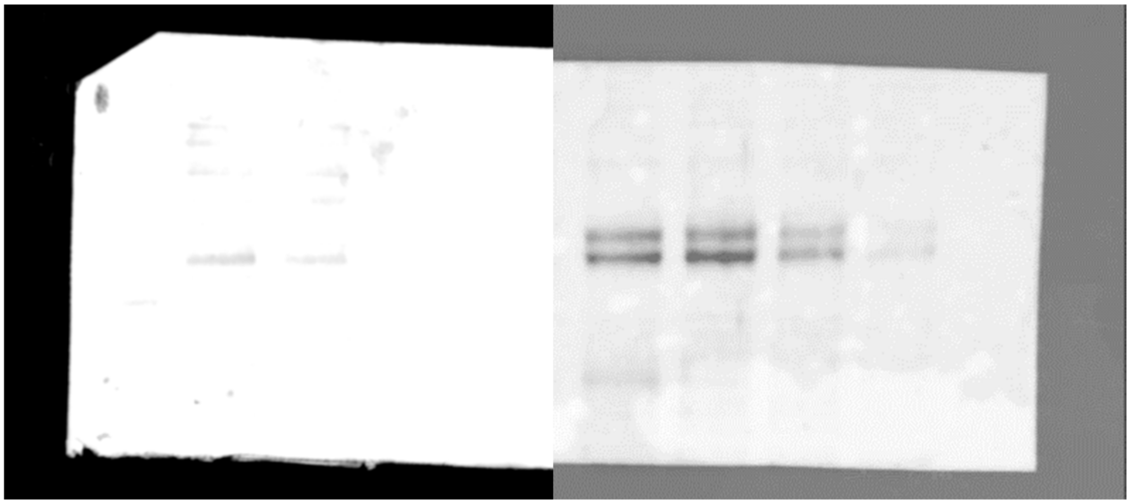

ICAM-1 blot (left: without TNF, right: with TNF)

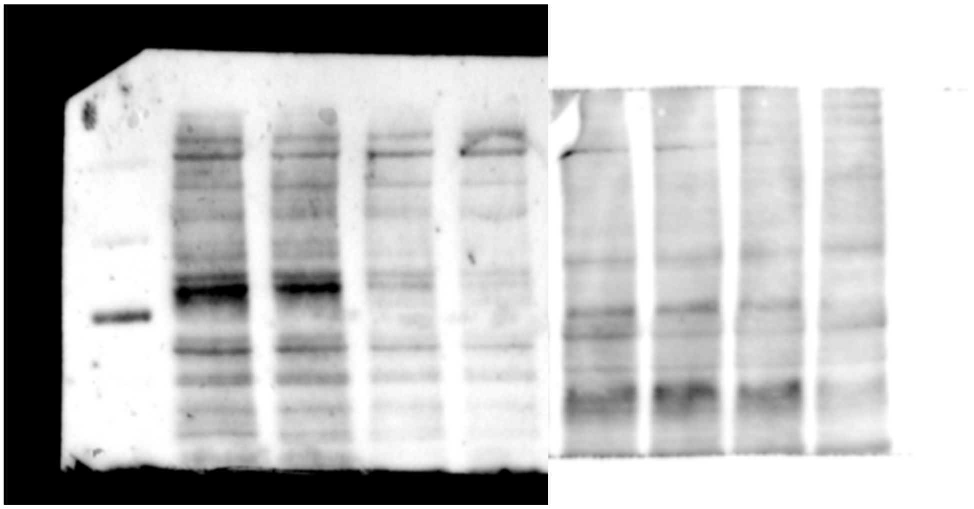

IRAK1-TRAF6 blot (left: without TNF, right: with TNF)

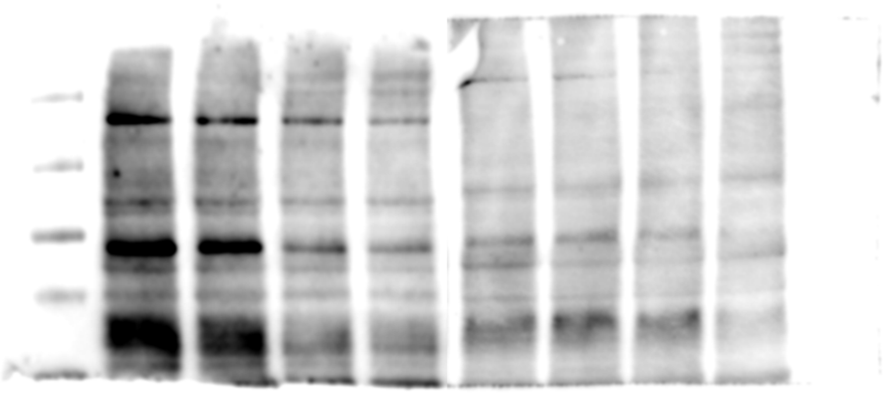

actin blot (left: without TNF, right: with TNF)

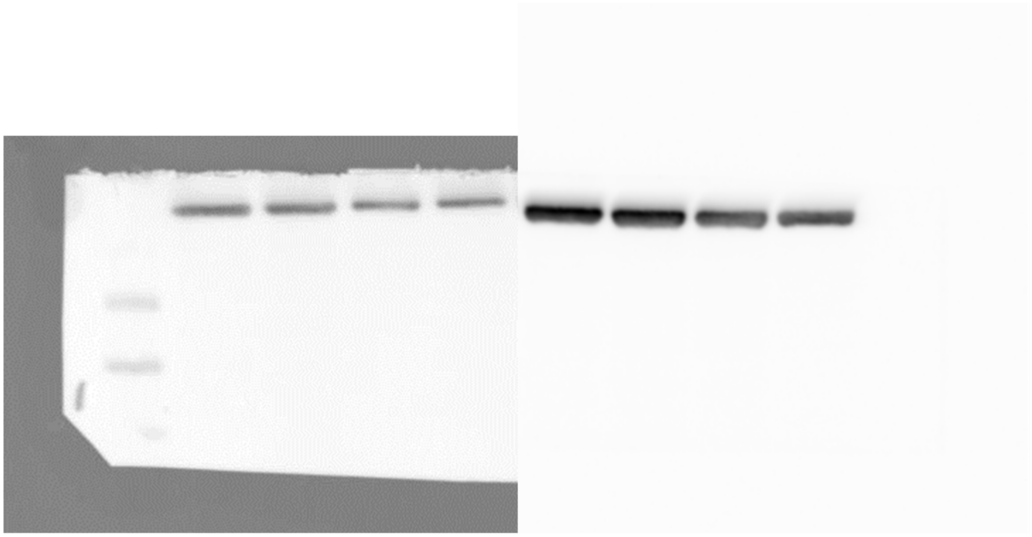

Supplement: Supplementary file 1 — Supplementary Information [file 41598_2019_44743_MOESM1_ESM.pdf]
